# Fluorescence polarization assay improves the rapid detection of human brucellosis in China

**DOI:** 10.1186/s40249-021-00834-3

**Published:** 2021-03-31

**Authors:** Shuai-Bing Dong, Di Xiao, Jing-Yao Liu, Hui-Mei Bi, Zun-Rong Zheng, Li-Da Wang, Xiao-Wen Yang, Guo-Zhong Tian, Hong-Yan Zhao, Dong-Ri Piao, Zhi-Feng Xing, Hai Jiang

**Affiliations:** 1 State Key Laboratory for Infectious Disease Prevention and Control, Collaborative Innovation Center for Diagnosis and Treatment of Infectious Diseases, National Institute for Communicable Disease Control and Prevention, Chinese Center for Disease Control and Prevention, Beijing, China; 2 Beijing Center for Disease Prevention and Control, Beijing Research Center for Preventive Medicine, Beijing, China; 3 General Hospital of Heilongjiang Province Land Reclamation Bureau, Harbin, China; 4 Heilongjiang Provincial Centre for Disease Control and Prevention, Harbin, China

**Keywords:** Human brucellosis, Fluorescence polarization assay, Diagnosis

## Abstract

**Background:**

Brucellosis is an infectious-allergic zoonotic disease caused by bacteria of the genus *Brucella*. Early diagnosis is the key to preventing, treating, and controlling brucellosis. Fluorescence polarization immunoassay (FPA) is a new immunoassay for relatively rapid and accurate detection of antibodies or antigens based on antigen–antibody interaction. However, there is no report on FPA-based detection of human brucellosis in China. Therefore, this study is to evaluate the value of FPA for the diagnosis of human brucellosis in China.

**Methods:**

We recruited 320 suspected brucellosis cases who had the clinical symptoms and epidemiological risk factors between January and December, 2019. According to China Guideline for Human Brucellosis Diagnosis, the Rose Bengal test (RBT) was used for the screening test, and the serum agglutination test (SAT) was used as the confirmatory test. Brucellosis was confirmed only if the results of both tests were positive. Additionally, FPA and enzyme linked immune sorbent assay (ELISA) were compared with SAT, and their sensitivity, specificity, coincidence rate and consistency coefficient (Kappa value) as diagnostic tests were analyzed individually and in combination. The optimal cut-off value of FPA was also determined using the receiver operator characteristic (ROC) curve.

**Results:**

The optimum cut-off value of FPA was determined to be 88.5 millipolarization (mP) units, with a sensitivity of 94.5% and specificity of 100.0%. Additionally, the coincidence rate with the SAT test was 96.6%, and the Kappa value (0.9) showed excellent consistency. The sensitivity and specificity of FPA and ELISA combined were higher at 98.0% and 100.0% respectively.

**Conclusions:**

When the cut-off value of FPA test is set at 88.5 mP, it has high value for the diagnosis of brucellosis. Additionally, when FPA and ELISA are combined, the sensitivity of diagnosis is significantly improved. Thus, FPA may have potential in the future as a diagnostic method for human brucellosis in China.

**Graphic abstract:**

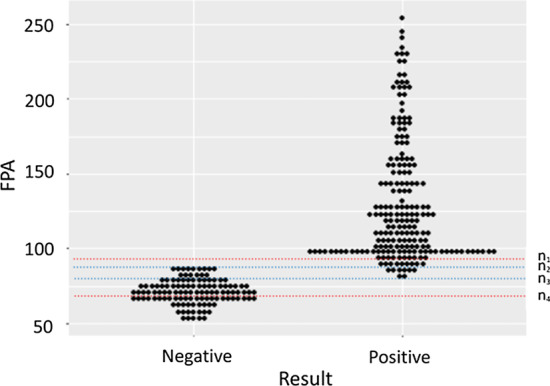

## Background

Brucellosis is an infectious-allergic zoonotic disease caused by bacteria of the genus *Brucella* [1]. The disease is transmitted to humans mainly by contact with infected animals and the ingestion of infected meat or unpasteurized dairy product [2]. Currently, more than 170 countries have reported human cases of brucellosis, and approximately 500 000 new cases are reported each year [3]. Despite this, brucellosis is a highly neglected zoonotic disease, according to the World Health Organization [4]. Brucellosis is especially prevalent in several low-income and middle-low-income countries [5]. In China, brucellosis has been recognized as an epidemic on account of its high incidence and wide spread since the mid-1990s, and it is an important public health problem in the country [6].

Early diagnosis is the key to preventing, treating, and controlling brucellosis. At present, there are many techniques for the detection of human brucellosis, but bacterial isolation and culture is still the gold standard [7]. However, this method has a low clinical isolation rate and is time consuming. Serological diagnosis of human brucellosis is usually easier and faster than bacterial isolation and culture, so serological diagnosis methods are widely used [8]. The Rose Bengal plate agglutination test (RBT) is a screening test, but its efficiency is greatly affected by the test conditions. Therefore, in China, an antiglobulin test (Coomb’s test) and serum agglutination test (SAT) are used as confirmatory tests, along with RBT for the diagnosis of brucellosis [9–11]. However, the procedures for these confirmatory tests are a bit complicated and time consuming, and the interpretation of the results is easily affected by subjective factors as, occasionally, false negatives occur due to the prozone phenomenon [12]. Based on the current situation, there is a clear need for more reliable tests for the diagnosis of brucellosis.

Fluorescence polarization immunoassay (FPA) is a new immunoassay for relatively rapid and accurate detection of antibodies or antigens based on antigen–antibody interaction [13]. FPA meets the standards of the World Organization for Animal Health, and, therefore, it has been adopted as a laboratory testing method for animal brucellosis. The advantages of FPA are that the reaction time is only 5 min and it can be used for both individual detection and large-scale field screening [14]. Further, unlike the conventional tests, data are obtained electronically. Therefore, any subjectivity is eliminated, and instead, rapid analysis, a permanent record, and easy data dispersal are possible. Some studies have reported that FPA is widely used to detect *Brucella* spp. antibody in the serum, whole blood, and milk of cattle [15], sheep [16], pigs [17], deer [18], camel [19], and other animals. There are also a few reports on the detection of human brucellosis with FPA [14, 20]. However, there is no report on FPA-based detection of human brucellosis in China. Therefore, in order to explore the possibility of applying FPA for human brucellosis diagnosis in China, in this paper, FPA was evaluated for its efficiency and compared with a variety of standard laboratory detection methods, including RBT, SAT, and enzyme linked immune sorbent assay (ELISA).

## Materials and methods

### Serum samples

This study included 320 patients with suspected brucellosis, who had the clinical symptoms of the disease and epidemiological risk factors. These patients were admitted to the Heilongjiang Provincial General Administration of Agriculture and Reclamation General Hospital between January 1, 2019, and December 31, 2019. Fasting venous blood (4 ml) was collected for brucellosis serological testing, and the diagnosis of brucellosis was based on the Diagnostic Criteria for Brucellosis WS269-2019 [21]. Suspected cases of brucellosis were defined as people with clinical symptoms [fever (≥ 37.5 ℃), fatigue, night sweats, and joint pain] and epidemiologic risk factors for infection. Confirmed cases were defined as suspected cases with an antibody titer of ≥ 1:100 (+ +) in SAT or positive *Brucella* isolate. If the antibody titer for SAT is 1:50, Coomb’s test is generally used as an additional confirmatory test. However, culture and isolation can be only performed at a few provincial-level laboratories and the National Brucellosis Laboratory in Beijing.

All subjects provided informed consent to participate in the study.

### Instruments

The following instruments were used: fluorescence polarimeter (FLUPO®, Peace River research Institute, Heilongjiang Province), fluorescence polarimetry test tube antibody detection kit (Peace River ®, 921,021, Peace River research Institute, Heilongjiang Province), ELISA antibody detection kit for brucellosis (Peace River®, 650,112, Peace River Research Institute, Heilongjiang Province), and RBT antigen and SAT antigen detection kits (BLSH-01 and BLSS-02, National Institute for Communicable Disease Control and Prevention, Chinese Center for Disease Control and Prevention).

### Detection methods

SAT, RBT, ELISA and Coombs were performed, and the data interpreted, according to the Diagnostic Criteria for Brucellosis WS269-2019 in China [21]. ELISA is used as a quantitative screening test for the diagnosis of brucellosis. If the serum OD ratio/positive control OD value is ≥ 24%, the patient is considered to be positive, and if it is < 24%, the patient is considered to be negative.

In FPA, the titer of antibody bound to the antigen directly is determined with the help of a fluorescent dye attached to a small antigen fragment, which is excited by plane polarized light of a specific wavelength. In the absence of an antibody, the molecular size of the antigen remains unchanged, and therefore, the rate of rotation and the extent of light polarization remains constant. On the other hand, when an antigen–antibody complex is formed, the molecular size increases. As a result, the rate of rotation is reduced and the extent of light polarization is high. This change can be measured by a fluorescence polarization analyzer, and the result is expressed in millipolarization (mP) units. According to the fluorescence polarization test tube antibody detection kit for brucellosis, the test result is negative when the FPA value is ≤ 72 mP; a value ≥ 93 mP indicates a positive result and a value between 72 and 93 mP indicates that there is a suspicion of brucellosis.

### Data analysis

Microsoft Excel software 2010 (Microsoft Office, CA, USA) and IBM SPSS Statistics 22.0 (IBM Corp; Armonk NY, USA) were used for data analysis. SAT was used as the confirmatory test. ELISA and FPA (individually and in combination) were compared with SAT, based on their sensitivity, specificity, coincidence rate, and consistency coefficient (Kappa value). A Kappa value ≤ 0.4 indicates poor consistency; 0.4 < Kappa < 0.75, medium and high consistency; and Kappa ≥ 0.75, excellent consistency. The optimal cut-off value of FPA was also determined using the receiver operator characteristic (ROC) curve.

## Results

### Demographic data and grouping

Of the 320 cases of suspected brucellosis, the results of both RBT and SAT were positive in 200 cases, which formed the brucellosis group. This group of 200 patients included 149 males and 51 females (mean age, 45.5 ± 13.4 years). The remaining 120 patients had negative results on both the RBT and SAT tests and served as the control group. This group included 70 males and 50 females, with a mean age of 39.8 ± 16.6 years (Table 1).

**Table 1:** Demographic features of brucellosis group and control group

Demographic feature	Brucellosis group *n* (%)	Control group *n* (%)
Sex		
Male	149 (74.5%)	70 (58.3%)
Female	51 (25.5%)	50 (41.7%)
Age group (years)		
< 20	4 (2.0%)	7 (5.9%)
20–30	26 (13.0%)	40 (33.3%)
31–40	33 (16.5%)	18 (15.0%)
41–50	64 (32.0%)	16 (13.3%)
51–60	46 (23.0%)	24 (20.0%)
> 60	27 (13.5%)	15 (12.5%)
Age (years), mean ± SD	45.5 ± 13.4	39.8 ± 16.6

*SD* Standard deviation

### FPA results

**Fig. 1:**
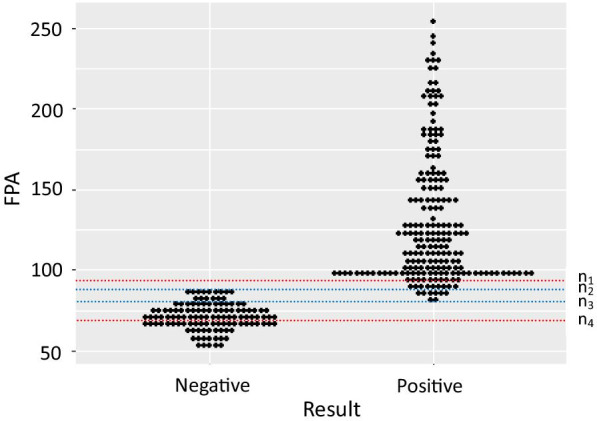
Interactive dot diagram of fluorescence polarization immunoassay (FPA) results for 200 confirmed (positive) cases of brucellosis and 120 control (negative) cases. n_1_ = 93 mP (reference standard upper limit for reagents). n_2_ = 88 mP (negative group maximum). n_3_ = 80 mP (positive group minimum). n_4_ = 72 mP (reference standard lower limit for reagents)

The FPA results showed that 180 patients were positive, 75 patients were suspicious for brucellosis, and 65 were negative. Among the 75 cases of suspected brucellosis, 20 were from the brucellosis group and 55 were from the control group (Table 2). The maximum FPA value of the negative group was 88 mP; the minimum FPA value of the positive group was 80 mP; the FPA values were between 80 and 88 mP in 20 out of 120 cases (16.7%) of the control group, and in 11 out of 200 cases (5.5%) in the brucellosis group (Fig. 1).

**Table 2:** Results of SAT and FPA summarized according to the reagent reference standard

SAT	FPA			Total
	+	Suspicious	−	
+	180	20	0	200
−	0	55	65	120
Total	180	75	65	320

*SAT* Serum agglutination test, *FPA* Fluorescence polarization immunoassay

### Optimal cut-off value for FPA

**Fig. 2:**
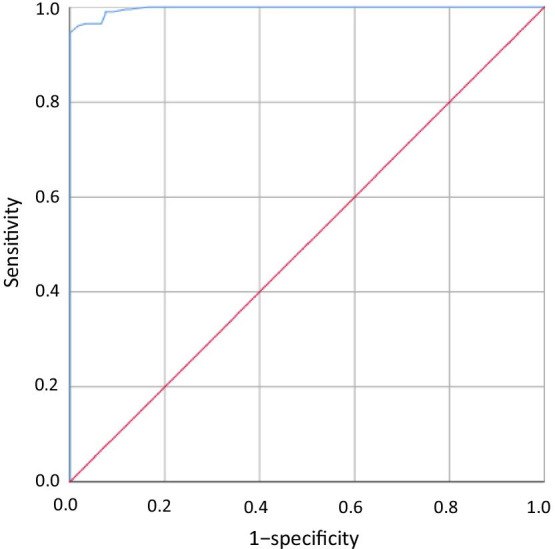
Receiver operator characteristic (ROC) analysis of sensitivity (%) plotted against 1 − specificity (%) to determine the optimal cut-off value of fluorescence polarization immunoassay (FPA) for the detection of antibodies against *Brucella* spp

At present, the FPA test is not included in the diagnostic criteria for brucellosis in China. Therefore, ROC curve analysis of the FPA test results was used to determine the optimal cut-off value. The results showed that the area under the curve was 0.997 [95% confidence interval (*CI*): 0.994–1.000, standard error: 0.002]. The optimal cut-off was determined as 88.5 mP, because it provided the maximum sum of sensitivity and specificity (194.5), with the individual sensitivity and specificity values being 94.5% and 100.0%, respectively (Fig. 2).

In 10 patients who were positive according to RBT and had a titer of 1:50 according to SAT, Coomb’s test was used to confirm the diagnosis of brucellosis. When the cut-off of 88.5 mP for the FPA test was applied in this group of patients, only one of the ten patients was found to be negative (as indicated by an FPA value less than 88.5 mP) (Table 3). Thus, there was only one false-negative result and no false-positive results with FPA.

**Table 3:** Serological results of SAT for patients with an antibody titer of 1:50 (*n* = 10)

Case	RBT	SAT (+ +)	Coomb’s (+ +)	ELISA (%)	FPA
12	+	50	200	17	85
25	+	50	200	24	128
52	+	50	200	23	109
72	+	50	200	71	144
78	+	50	200	18	90
104	+	50	200	22	142
105	+	50	200	15	113
121	+	50	200	13	96
133	+	50	200	20	98
199	+	50	200	42	145

*SAT* Serum agglutination test, *RBT* Rose bengal test, *ELISA* Enzyme linked immunesorbent assay, *FPA* Fluorescence polarization immunoassay, + Positive; +  + Antibody titer

### Comparison and combination of FPA and ELISA

For the FPA test results, based on the determined cut-off value, an FPA value of ≥ 88.5 mP was considered to be positive, and a value of < 88.5 mP was considered to be negative. With SAT as the reference test, the sensitivity, specificity, and coincidence rate of FPA, ELISA, and FPA combined with ELISA were analyzed. The sensitivity, coincidence rate, negative predictive value, and Kappa value of the FPA test were higher than those of the ELISA test. The sensitivity and specificity of the FPA and ELISA tests combined were as high as 98.0 and 100.0%, respectively (Table 4).

**Table 4:** Analysis of the results of SAT, FPA, and ELISA

SAT	FPA			ELISA			FPA combined with ELISA			Total
	+		−	+		−	+		−	
+	189		11	161		39	196		4	200
−	0		120	0		120	0		120	120
Sensitivity (%)		94.5			80.5			98.0		
Specificity (%)		100.0			100.0			100.0		
Coincidence rate (%)		96.6			87.8			98.8		
PPV (%)		100.0			100.0			100.0		
NPV (%)		91.6			75.5			96.8		
Kappa		0.9			0.8			1.0		

*PPV* positive predictive value, *NPV* negative predictive value, *SAT* Serum agglutination test, *ELISA* Enzyme linked immunesorbent assay, *FPA* Fluorescence polarization immunoassay, + Positive, − Negative

## Discussion

In this study, we have assessed the efficiency of FPA for the diagnosis of human brucellosis in China. FPA was compared to other tests and also combined with ELISA to determine its efficiency. Additionally, the optimal cut-off value of FPA for this population was also determined.

In the present study, the ROC curve analysis for the FPA results showed that when the cut-off value is 88.5 mP, the sensitivity and specificity of the FPA test are at an optimum, at 94.5% and 100.0%, respectively. Cut-off values for FPA have also been reported by Konstantinidis in a Greek population [20]. The optimum cut-off value reported was 99 mP, and the sensitivity and specificity were 93.5% (95% *CI*: 89.5–96.3) and 96.1% (95% *CI*: 93.2–97.9) respectively. In a similar study in Argentina, Lucero [14] reported an optimal cut-off value of 72 mP, with a sensitivity and specificity of 96.1% and 97.9% respectively. The difference in sensitivity across these studies and the present one might be related to differences in the study populations tested. The dada from Dr. Lucero showed that the positive cases were culture-proven population, while in this study, the positive of both RBT and SAT tests were used for confirmed cases of the human brucellosis, according to the Diagnostic Criteria for Brucellosis WS269-2019 in China [21].

In the present study, FPA was found to have excellent consistency (Kappa value = 0.93), and the coincidence rate with the SAT test was 96.6%. However, there were 11 false-negative cases. This might have been caused by poor affinity of the antigen with the antibody or a low titer of serum antibodies [20]. If the cut-off value is decreased, the sensitivity may increase, but the specificity will certainly decrease. Alternatively, FPA could be combined with ELISA, as the sensitivity of brucellosis diagnosis was improved with FPA and ELISA combined, according to the present findings.

When FPA and ELISA were compared in this study, the sensitivity, coincidence rate, negative predictive value, and Kappa value of FPA were found to be higher than those of ELISA. Studies have shown that ELISA has a sensitivity of 83.3% for IgM and 41.7% for IgG, while the combined specificity for IgG and IgM is 92.3% [22]. Therefore, the present comparison might have been affected by the disease course of the patient. Nonetheless, there was no false-positive result with FPA. The lack of false-positive results indicates that there was no cross-reaction of antibodies produced by bacteria with structurally similar antigens (such as *Yersinia enterocolitis* O:9, *Escherichia coli* O:157, *Salmonella* serotypes of Kaufmann-White group N, and *S. maltophilia*) [23]. The finding is in basically agreement with the report of Nielsen about cross-reaction [13]. Therefore, given that Coomb’s test is complicated and time consuming, the FPA test could potentially replace Coomb’s test based on the present findings.

**L**imitation of this study is that bacterial culture was not performed for confirmation of the results, even though isolation of *Brucella* spp. from blood, tissue or bone marrow cultures is known to be the only means of definitively diagnosing brucellosis [21]. Despite this, at the cut-off value of 88.5 mP, FPA has high sensitivity and specificity. Additionally, FPA is rapid, convenient, and reliable as a quantitative test. The results also show that when FPA is combined with ELISA, the sensitivity of brucellosis diagnosis can be significantly improved. In the future, the reproducibility of FPA test should be determined, so that it can be used for screening and confirmation of brucellosis in China.

## Conclusions

FPA is a new immunoassay for relatively rapid and accurate detection of antibodies or antigens. When the cut-off value of FPA test is set at 88.5 mP, it has high value for the diagnosis of brucellosis. Additionally, when FPA and ELISA are combined, the sensitivity of diagnosis is significantly improved. Thus, FPA may have potential in the future as a diagnostic method for human brucellosis in China.

## Data Availability

All original (de-identified) data and materials are available upon request from the corresponding author.

## Abbreviations

*CI*: Confidence interval
ELISA: Enzyme linked immune sorbent assay
FPA: Fluorescence polarization immunoassay
mP: Millipolarization
NPV: Negative predictive value
PPV: Positive predictive value
RBT: Rose bengal test
ROC: Receiver operator characteristic
SAT: Serum agglutination test
SD: Standard deviation
